# Silica Gel Coated Spherical Micro resonator for Ultra-High Sensitivity Detection of Ammonia Gas Concentration in Air

**DOI:** 10.1038/s41598-018-20025-9

**Published:** 2018-01-26

**Authors:** Arun Kumar Mallik, Gerald Farrell, Dejun Liu, Vishnu Kavungal, Qiang Wu, Yuliya Semenova

**Affiliations:** 1 Photonics Research Centre, Dublin Institute of Technology, Kevin Street, Dublin, 8 Ireland; 2 Department of Mathematics, Physics and Electrical Engineering, Northumbria University, Newcastle Upon Tyne, NE1 8ST United Kingdom

## Abstract

A silica gel coated microsphere resonator is proposed and experimentally demonstrated for measurements of ammonia (NH_3_) concentration in air with ultra-high sensitivity. The optical properties of the porous silica gel layer change when it is exposed to low (parts per million (ppm)) and even ultra-low (parts per billion (ppb)) concentrations of ammonia vapor, leading to a spectral shift of the WGM resonances in the transmission spectrum of the fiber taper. The experimentally demonstrated sensitivity of the proposed sensor to ammonia is estimated as 34.46 pm/ppm in the low ammonia concentrations range from 4 ppm to 30 ppm using an optical spectrum analyser (OSA), and as 800 pm/ppm in the ultra-low range of ammonia concentrations from 2.5 ppb to 12 ppb using the frequency detuning method, resulting in the lowest detection limit (by two orders of magnitude) reported to date equal to 0.16 ppb of ammonia in air. In addition, the sensor exhibits excellent selectivity to ammonia and very fast response and recovery times measured at 1.5 and 3.6 seconds, respectively. Other attractive features of the proposed sensor are its compact nature, simplicity of fabrication.

## Introduction

Ammonia (NH_3_) and its compounds are used in the production of liquid fertilizers, in refrigeration, during manufacturing of dyes, drugs, synthetic fibers, in food processing and biological sciences^1,2^. At room temperature, ammonia is a colorless, highly corrosive and toxic gas with pungent odor. Exposure to high concentrations of ammonia in air causes a burning sensation in the nose, throat and respiratory track. The safe limit of NH_3_ concentration that people may work in for more than 8 hours is set to be 25 ppm and 35 ppm for a shorter duration^2^. These thresholds are below the detection limit of a human’s sense of smell and therefore some means to sense and also quantify ammonia concentration in the air is needed. Furthermore, sensors with detection thresholds in the order of ppb are very valuable. In the semiconductor industry for example, deep ultraviolet photo resist is extremely sensitive to the presence of airborne molecular gases inside a clean room, where even the presence of ammonia with a concentration of only 17 ppb for 10 minutes will deteriorate the performance of the lithography process^3^. Optical fiber based sensors have some unique advantages over their electrical equivalents such as remote and real time monitoring, immunity to various sources of disturbances e.g. electromagnetic interference, radioactivity and explosive environments which makes them a cost-effective, flexible and inert sensing solution. Many optical fiber based ammonia sensors have been reported to date, based on different operating principles and with different sensitivities. A surface plasmon resonance based fiber optic ammonia gas sensor has been proposed in^4,5^. Adolfo *et al*. successfully demonstrated sensing of ammonia concentrations of up to 100 ppm using bromocresol, a pH indicator, attached to an optical fiber^6^. Cao *et al*. also used bromocresol polymer to generate an evanescent field in a U-shaped plastic clad silica multi-mode fiber to sense ammonia^7^.

Many of the existing ammonia sensors have been designed to realize detection in the order of ppm, but those are unable to reach ppb level detection, required by some applications. Sensors based on the whispering gallery mode (WGM) effect in spherical microresonators have found many applications for very sensitive detection of molecular adsorption^8^, refractive index^9^, temperature^10^ and gas concentration^11^ due to their ultra-high quality factors (Q), low absorption losses and inexpensive fabrication. WGMs are high angular momentum modes which can be excited by trapping light propagating inside a dielectric structure with a circular symmetry, such as a microsphere, by repeated total internal reflections.The spectral positions of WGM resonances are strongly dependent on the geometry of the dielectric resonator (diameter, sphericity), the optical properties of the resonator material and also on the refractive index (RI) of surrounding the resonator medium. WGMs in a silica micro sphere can be excited by trapping a portion of evanescent field propagating through a fiber taper and the changes in the surrounding RI can be measured by monitoring either reflected or transmitted light through the taper. Recently, we proposed and demonstrated a highly sensitive relative humidity (RH) sensor based on the similar microsphere coated with a thin layer of agarose hydrogel prepared from a 2.25% wt./vol. agarose solution^12^.

In this paper, we propose and experimentally demonstrate a silica gel coated microsphere for the detection of ammonia vapor concentration in air at a constant relative humidity. The silica microsphere is fabricated at the tip of a short length of SMF-28 fiber and a single layer of silica gel is deposited on the surface of the microsphere by dip coating. WGMs in the microsphere are excited by evanescent coupling of light using an adiabatic fiber taper with a waist diameter of approximately 3–4 *μ*m. Exposure of the silica gel coating layer to ammonia vapor results in changes in its refractive index leading to the corresponding shifts of the WGM resonances. A detailed investigation of the sensor performance has been carried out by its exposure to low concentrations as well as ultra-low concentrations of ammonia vapor inside the test chamber at constant humidity 50%RH and constant temperature 23 °C conditions.

## Methods

### Preparation of porous silica gel

Silica gel in laboratory conditions is typically prepared by sol-gel polymerization of tetra-ethylorthosilicate (TEOS) under hydrolic conditions using either acids or base catalysis. For our experiments, tetraethylorthosilicate (TEOS), ethyl alcohol and sulphuric acid (H_2_SO _4_) were purchased from Sigma Aldrich and were used without any further purification. 10 mL of TEOS and 5 mL of ethanol were mixed in a 250 mL measuring flask and kept in a magnetic stirrer for 20 minutes at room temperature. 1.5 mL of 0.1 mol/L H_2_SO _4_ was poured into the TEOS solution in the flask under constant stirring. The solution turned to a gel form after 3 hours of continuous stirring.

### Microsphere resonator fabrication

**Figure 1:**
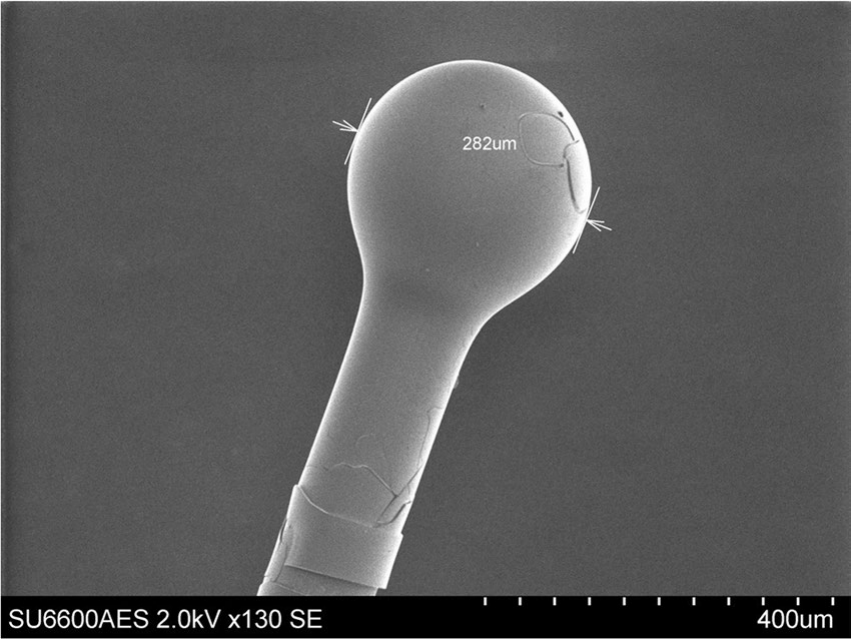
SEM image of porous silica coated microsphere used in experiment.

The microsphere used for our experiments was fabricated at the tip of a standard single mode fiber by discharging a series of electric arcs. The fiber tip was gradually melted by the arcs and assumed a spherical shape due to surface tension. The sphere diameter was controlled by the number of arcs induced on the tip of the fiber. The fabricated microsphere was then dipped into the silica gel and pulled out very fast. Coated with the porous silica gel, the microsphere was then dried at room temperature for 24 hours. Fig. 1 shows a scanning electron microscope (SEM) image of the coated microsphere used in our experiment. From Fig. 1 the sphere diameter is estimated as 282 *μm*. To allow for coupling of light to and from the microsphere, an adiabatic tapered fiber was fabricated using a customized micro heater brushing technique^13^. In our experiment the tapered waist diameter was approximately 3–4 microns. To improve mechanical stability, the fabricated fiber taper was placed in direct contact with the microsphere and fixed on a glass slide at a height of 5 mm from the slide surface using two drops of UV curable epoxy (Norland).

### Calculation of vapor concentrations

Ammonia, humidity and other gases sensing experiments were carried out in a humidity and temperature-controlled chamber with a volume of 106 L. In general for gas vapour sensing experiments, the control of the vapor concentration was achieved by injecting a certain volume of liquid analyte into a test chamber with a known volume using a micro syringe. The resulting concentration of the vapor is calculated using the following equation^14^.

1 $${C}_{ppm}=\frac{{V}_{\mu L}\times {D}_{gm{L}^{-1}}}{{M}_{gmo{l}^{-1}}\times {V}_{mL}}\times 2.42\times {10}^{7}$$

where, *C*_*ppm*_ is the required vapor concentration,*V*_*μL*_ is the volume of the liquid analyte, ${D}_{gm{L}^{-1}}$ is the density of the liquid, *V*_*mL*_ is the volume of the chamber and ${M}_{gmo{L}^{-1}}$ is the molecular weight of the liquid analyte. All the subscripts are the corresponding units of measurement. The temperature and humidity inside the test chamber were maintained constant at 23 °C and 50% RH, respectively throughout the ammonia and volatile organic compounds (VOCs) experiments.

### Two methods for characterization of the sensor performance

**Figure 2:**
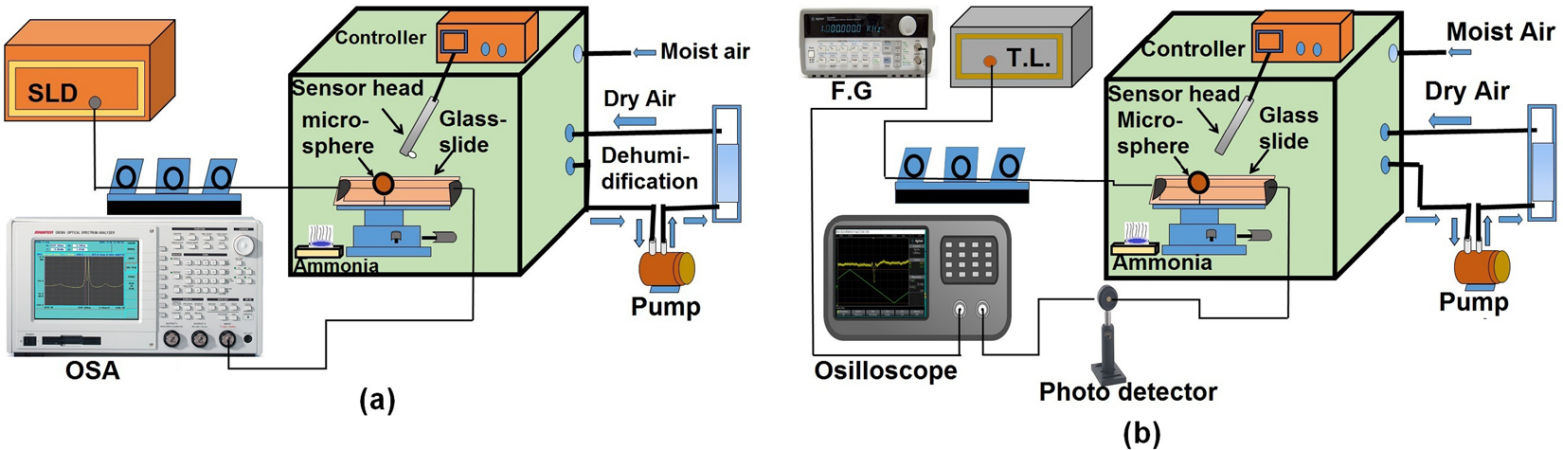
Experimental setup for ammonia sensing using (**a**) OSA interrogation system; (**b**) frequency detuning method.

The characterization of the proposed ammonia gas sensor was carried out using two different interrogation methods. In one of the methods we used a superluminescent (SLD) light source (Thorlabs S5FC1005S), a polarization controller and an optical spectrum analyzer (OSA, Advantest Q8384) to measure the total WGM resonance wavelength shift against the wide range of low concentrations of ammonia from 4 ppm to 30 ppm as shown in Fig. 2(a). For concentrations below 4 ppm, a frequency detuning method was used, with the corresponding experimental setup shown in Fig. 2(b), consisting of an external cavity laser (Tunics Plus, linewidth 300 kHz, with operating wavelength range 1490–1640 nm) and a photo detector connected to high speed digital oscilloscope (Agilent MSOX-2022A) to record the WGM spectrum.

In the frequency detuning method the tunable laser was swept finely and continuously within 3 GHz across the selected WGM resonance with the help of a sawtooth current modulation realized by the function generator (F.G). The former interrogation method has a wider dynamic range for measurements with a limited resolution while the later (frequency detuning) method allows for a higher measurements resolution in a narrow range. In both experimental setups, the sensor sample - a coated micro-sphere, was mounted on an XYZ nano-positioning stage and then slowly brought in contact with the tapered fiber inside the humidity chamber as shown in Fig. 2. In both cases the temperature and moisture inside the chamber were controlled by the chamber’s controller system (ETS 5503).

## Results

### Experimental investigation of the sensor in a wider range of low (>4–30 ppm) ammonia concentrations using an OSA

**Figure 3:**
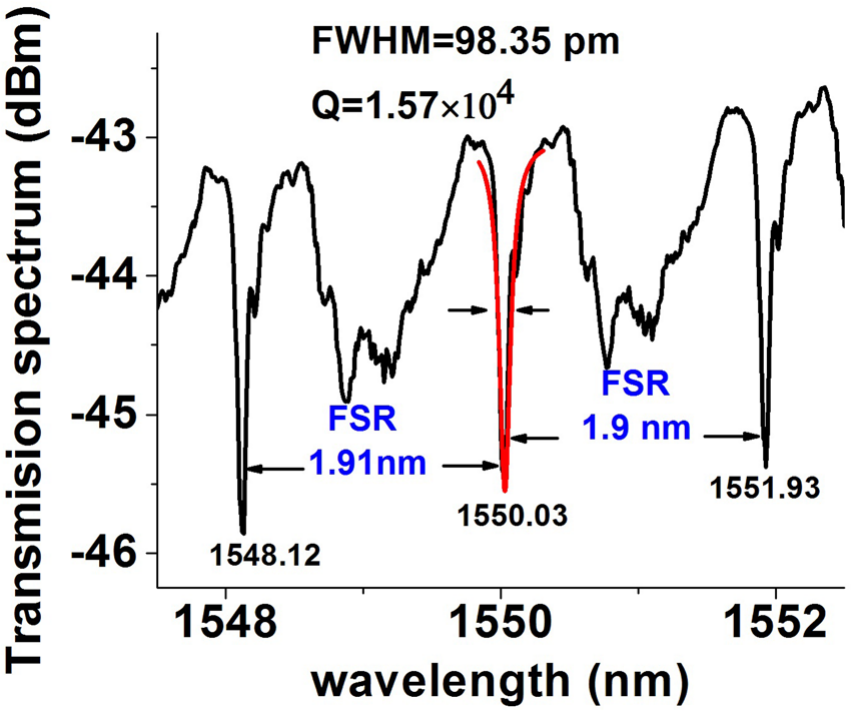
Transmission spectrum recorded by the OSA for a coated microsphere with a 282 *μm* diameter.

In the optical setup shown in Fig. 2(a) the light from the broadband SLD was launched into one of the ends of the fiber taper and the corresponding transmission spectrum was observed at the output end by means of an OSA. The wavelength resolution of the OSA was 10 pm. The microsphere was gradually and carefully brought in direct contact with the tapered fiber until the WGM resonances were clearly observed in the transmission spectrum of the fiber taper. The input light polarization was adjusted using a manual polarization controller to achieve maximum light coupling efficiency. Figure 3 illustrates a typical transmission spectrum recorded with the OSA. As can be seen from Fig. 3, periodic transmission dips with a quality factor (Q) in the order of 10 ^4^ and the free spectral range (F.S.R) of 1.9 nm can be clearly observed.

**Figure 4:**
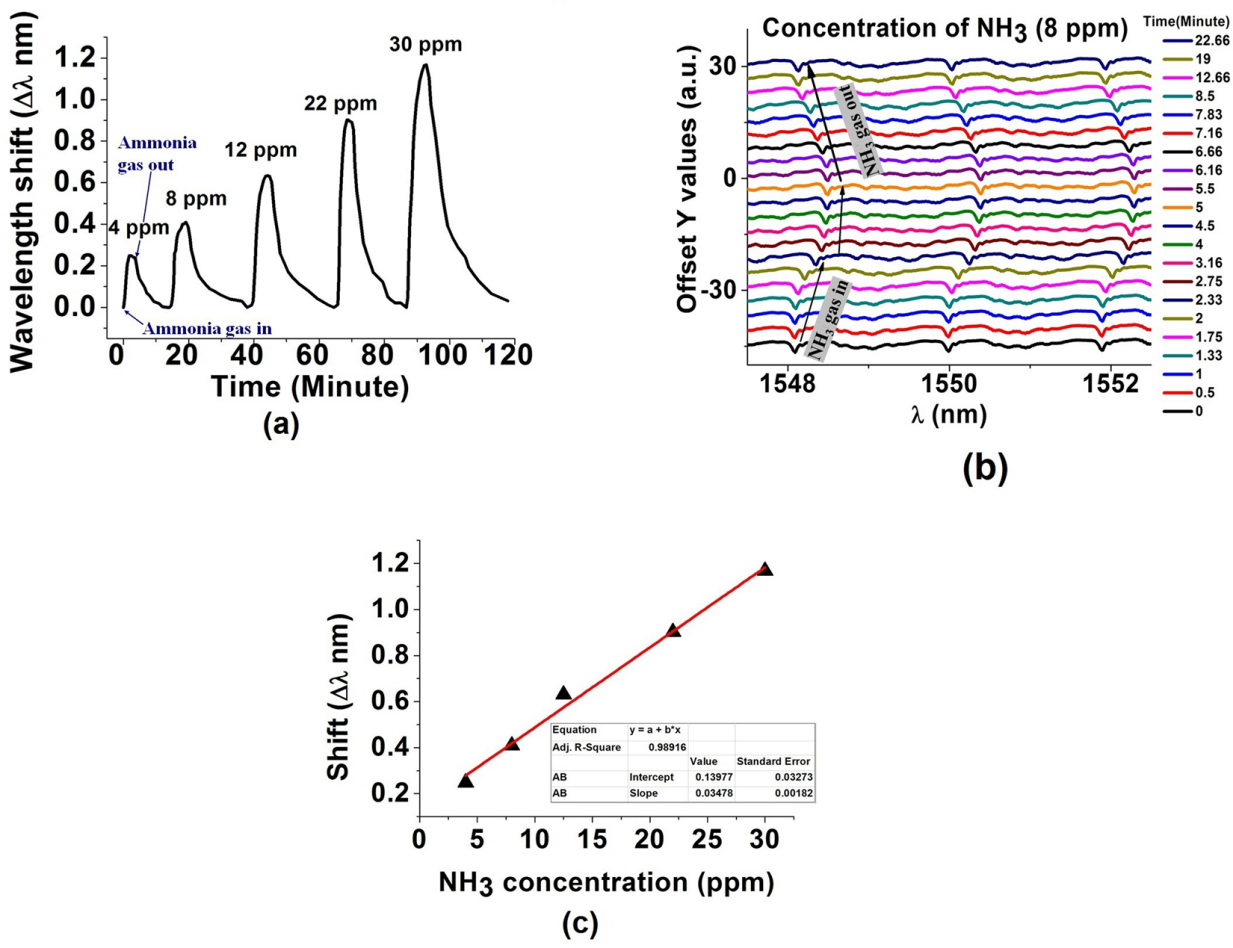
(**a**) WGM spectral shift during sensor’s exposure to different concentrations of NH_3_ ranging from 4 ppm to 30 ppm. (**b**) Sensor’s temporal response to the ammonia concentration of 8 ppm. Inset figure illustrates the wavelength shift for a selected WGM resonance in response to exposure to 8 ppm concentration of ammonia. (**c**) Sensor’s response as a function of NH_3_ concentration.

To investigate the response of the porous silica coated microsphere to ammonia vapors with different concentrations, small amounts of ammonium hydroxide (NH_4_OH) liquid were injected into and removed from the gas chamber in sequence, in order to create several low concentrations corresponding to 4 ppm, 8 ppm, 12 ppm, 22 ppm and 30 ppm of ammonia in air. As shown in Fig. 4(a), the WGM resonant wavelengths experience a red shift when the sensor is exposed to ammonia vapors. The spectral response of the sensor to an 8 ppm concentration of ammonia is summarized in Fig. 4(b), where multiple WGM spectra are compiled on a single graph to illustrate their shift in time after the sensor was exposed to the ammonia vapor. The main reason behind the shift is a change in the effective refractive index of the silica coating when NH_3_ molecules are adsorbed on the surface of the sphere. A higher concentration of NH_3_ leads to a larger red shift in the WGM spectrum. When the gas is removed from the chamber by opening an exhaust valve, the resonance wavelengths recover to their original positions. The maximum spectral shift of the WGM resonances in response to a certain ammonia concentration is plotted as a function of ammonia concentration in Fig. 4(c) and is found to be close to to linear with sensitivity estimated as 34.8 pm/ppm. It is important to note that the measured time response is not that of the sensor alone but also includes the time taken for the vapor concentration to stabilize inside the chamber.

### Experimental investigation of the sensor in the ultra-low ammonia concentrations range using frequency detuning method

**Figure 5:**
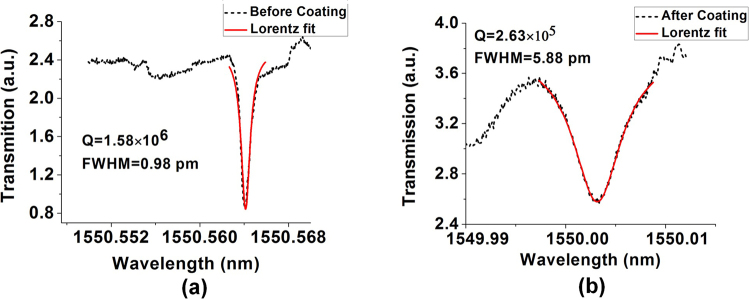
Q factor estimates for the 282 *μ*m diameter silica sphere (**a**) before and (**b**) after its coating with porous silica gel.

The output spectrum of the proposed sensor has a high Q-factor, typical for many WGM based sensors, potentially providing the capability to detect ultra-small concentrations of vapor phase ammonia inside the chamber. However the relatively low resolution of the OSA employed in the experiments described above means that we cannot fully utilise the high Q-factor available and in turn this limits the ability of the sensor to measure ultra-small concentrations of ammonia vapor. To overcome this issue many researchers employ a tunable laser with frequency detuning method to characterize WGM resonators^15–18^. A schematic diagram of our experimental setup employing the frequency detuning method is shown in Fig. 2(b). The transmitted power data was recorded as a function of time using a customized LabVIEW program. The Q-factor of a typical WGM resonance for the same coated microsphere and tapered fiber system, determined using the frequency detuning setup was calculated as 1.58 × 10^6^ using the formula

2 $$Q=\frac{{\lambda }_{R}}{{\rm{\Delta }}{\lambda }_{FWHM}}$$

where *λ*_*R*_ is the resonance wavelength and Δ*λ*_*FWHM*_ is the full width at half-maximum of the Lorentz function before coating (Fig. 5(a)). After coating the microsphere with silica gel, its Q-factor is reduced to 2.63 × 10^5^ as a result of increasing absorption and surface scattering losses in the coating layer (Fig. 5(b)).

**Figure 6:**
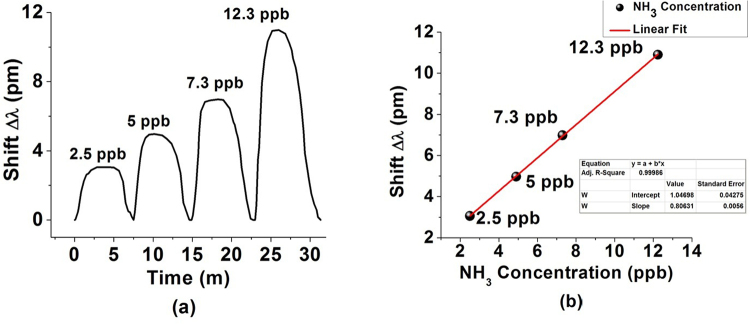
(**a**) Wavelength shifts of the WGM resonance versus time during the sensor’s exposure to various vapor phase ammonia concentrations. (**b**) A linear fit of the wavelength shift data.

Four different ammonia solutions with ultra-low concentrations of 2.5 ppb, 5 ppb, 7.3 ppb and 12.3 ppb were prepared using the method described in Equation (1) and a series of experiments was carried out to characterize the sensor’s response. Similar to the previous experiment, WGM resonances show a red shift in response to the presence of ultra-low concentrations of ammonia and remain stable during the time of exposure. After opening the chamber exhaust door, the WGM spectrum returns to its original position. The temperature and humidity inside the chamber were kept constant throughout the experiments. Figure 6(a) illustrates the shift of the WGM spectrum in response to a series of injections of ultra-low concentrations of ammonia vapor. The linear regression value R^2^ = 0.99986 illustrates the high linearity of the sensor’s response within the range from 2.5 ppb to 12.3 ppb of ammonia vapor concentrations as shown in Fig. 6(b). The average sensitivity of the sensor within the studied range of concentrations is estimated as 800 pm/ppm.

## Discussion

**Figure 7:**
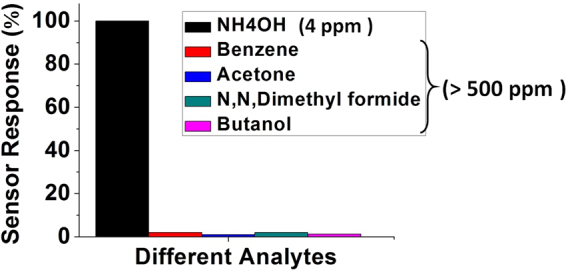
Sensor’s response to various volatile organic compounds.

To investigate the selectivity of the sensor’s response towards ammonia, similar experiments were carried out with other gases generated by volatile liquids such as acetone, methanol, benzene and n, n-dimethyl formide, and the corresponding results are presented in Fig. 7.

The diagram in Fig. 7 graphically compares the sensor’s response (maximum wavelength shifts) for the different vapor types expressed as a percentage of the response to a 4 ppm concentration of ammonia. As one can see from the diagram, the concentration of NH_3_ vapor as low as 4 ppm results in a much stronger response, whereas the other vapors require a much higher (>500 ppm) concentration to achieve a much smaller response. Thus it can be concluded that the silica gel coated microsphere resonator exhibits a high selectivity to ammonia and can be considered as an excellent optical sensor for detection of ammonia vapor even in the presence of other gases.

**Figure 8:**
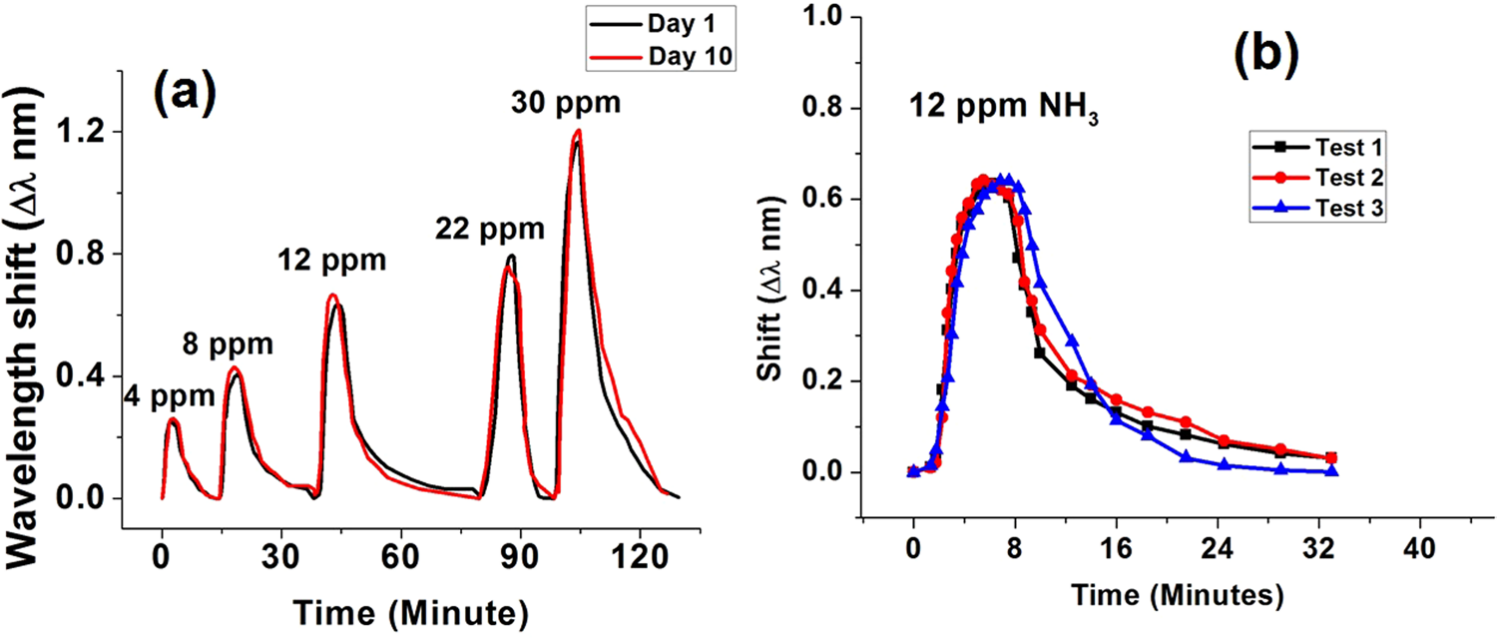
(**a**) Time responses of the sensor to NH_3_ recorded with a 10-days interval. (**b**) Sensor’s responses to 12 ppm of ammonia during three consecutive tests at constant humidity and temperature.

Stability and repeatability of performance are two important characteristics influencing practical applications of any optical sensor.To test the longer-term stability of the sensor’s response, ammonia sensing experiments, using an SLD light source and an optical spectrum analyzer, were carried out for the same sensor sample with a time interval of 10 days. In both experiments the concentrations of ammonia were 4 ppm, 8 ppm, 12 ppm, 22 ppm and 30 ppm at a constant temperature of 23 °C and 50% RH. It can be seen from Fig. 8(a), that the performance of the sensor is very stable within 10 days period with only small fluctuations.In addition to the stability tests, the sensor was tested for repeatability by exposing it to 12 ppm of ammonia under the same environmental conditions inside the chamber three times consecutively. Figure 8(b) illustrates the results of the repeatability study and shows that the value of the wavelength shift was almost same in each test.

**Figure 9:**
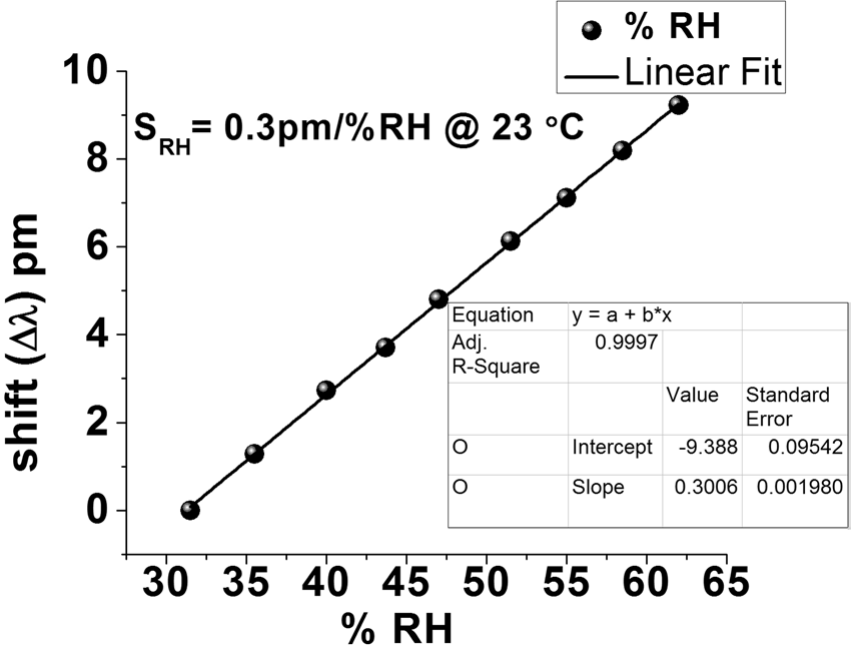
WGM spectral shift versus RH change from 30–65%RH silica gel coated on 282 *μm* microsphere at 23 °C.

Due to the porous nature of the silica gel coating and its excellent ability to adsorb water, the sensor can also be used to measure the concentration of water vapor in air^19–21^. To study the sensitivity of our sensor to relative humidity (RH) we carried out experiments using the same setup, shown in Fig. 2(b). The airtight test chamber shown in the figure is equipped with an inlet and an outlet. The upper inlet is made for the entry of the moist air into the chamber and the outlet is used for dragging the moist air to the dehumidifier through a pump. The dehumidifier box contains anhydrous calcium sulfate which absorbs moisture from the air. The compressed dry air is pumped in to regulate the RH in the chamber. Humid air was obtained by passing dry air through an ultrasonic humidification system (ETS5462). Controlling the ratio of humid air and compressed dry air in the chamber gives a fixed RH level. The chamber controller system allows for the independent setting of both temperature and relative humidity inside the chamber. The accuracy of the chamber is ±2% RH with the reference electronic humidity sensor resolution of 0.1% RH. Each humidity measurement was recorded five minutes after the RH level reached a set value to allow for the humidity throughout the chamber to stabilize. The sensor response was recorded using the the setup shown in Fig. 2(b) for a range of RH values from 30% to 65% RH. The corresponding results for the 282 *μ*m diameter microsphere coated with silica gel are shown in Fig. 9. As can be seen from the figure, an increase in the surrounding RH leads to a red shift of the WGM spectrum. This could be explained by the fact that when the surrounding RH increases, the silica coating adsorbs more water from the environment within the air pores which leads to an increase of the effective refractive index of the coating and the microresonator overall. The corresponding RH sensitivity values (S_*RH*_) estimated from the graph are 0.3 pm/% RH with high linearity characterized by the regression value of R ^2^ = 0.9997.

**Figure 10:**
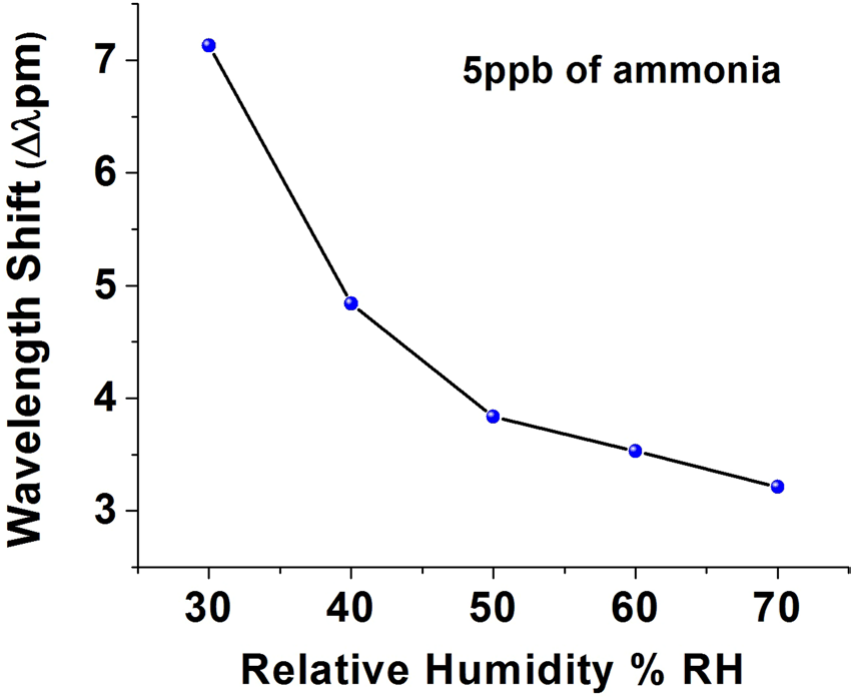
WGM spectral shift in response to 5 ppb of ammonia at different RH levels for a silica gel coated 282 *μm* diameter microsphere at constant temperature of 23 °C.

Sensor’s response to ammonia gas at different relative humidity levels was also investigated and the result is shown in Fig. 10. The graph illustrates the resonant wavelength shift due to exposure to 5 ppb of ammonia at different humidity values in the range from 30% RH to 70% RH at constant temperature inside the chamber. Frequency detuning method was used to record the data. As can be seen from the figure, response of the proposed sensor to ammonia gas decreases gradually with increase of humidity, possibly due to the increased competition from the water molecules being adsorbed on the porous silica surface, thus reducing the number of adsorbed ammonia molecules.

The detection limit (DL) is a vital parameter used to quantify the device sensing capability. The detection limit represents the smallest measurable physical parameter change and it can be expressed as^22^:

3 $$DL=\frac{R}{S}$$

where R is the resolution of the sensor and S is the sensor sensitivity. R can be calculated as

4 $$R=\sqrt{{\sigma }_{N}^{2}+{\sigma }_{T}^{2}+{\sigma }_{SR}^{2}}$$

where *σ*_*N*_, *σ*_*T*_,and *σ*_*SR*_ represent the standard deviations of the random effects associated with amplitude noise, temperature and detector spectral resolution, respectively. The estimates of DL for both detection methods used in our experiments are explained in Table 1. The corresponding standard deviations in the table were calculated in accordance with reference^22^. The SNR (Signal-to-Noise Ratio) of the OSA and the frequency detuning system where determined from specification and by calculation respectively are approximately 60 dB resulting in a *σ*_*N*_ calculated as 0.694 pm for the OSA and as 0.041 for the frequency detuning method. The standard deviations due to temperature stabilization for both techniques are taken as *σ*_*T*_ = 0.01 pm.The error in determining the position of the resonant mode is uniformly distributed between −0.3 pm and + 0.3 pm and the resulting standard deviation associated with the spectral resolution of the broadband source used with the OSA is *σ*_*SR*_ = 0.1732 pm. In the case of the tunable laser, the spectral deviation of the laser with a line width less than 1 MHz is estimated as *σ*_*SR*_ = 0.001 pm. Hence, the ammonia DL for the proposed sensor is calculated as 62 ppb when using the OSA method, and as 0.16 ppb when using the frequency detuning method for the same coated sphere sensor sample. Similarly, the DL for humidity measurement is estimated as 4.2 × 10^−1^ RH in the humidity range of 30–65% RH.

**Table 1:** Detection limit calculation for the silica gel coated microsphere sensor.

Parameters	Detection Technique	
	OSA	Frequency detuning
*σ*_*N*_(pm)	0.694	0.041
*σ*_*SR*_(pm)	0.173	0.001
*σ*_*T*_(pm)	0.01	0.01
S (pm/ppm)	34.8	800
R (pm)	2.145	0.128
DL(ppb of NH_3_)	**62**	**0.16**

**Figure 11:**
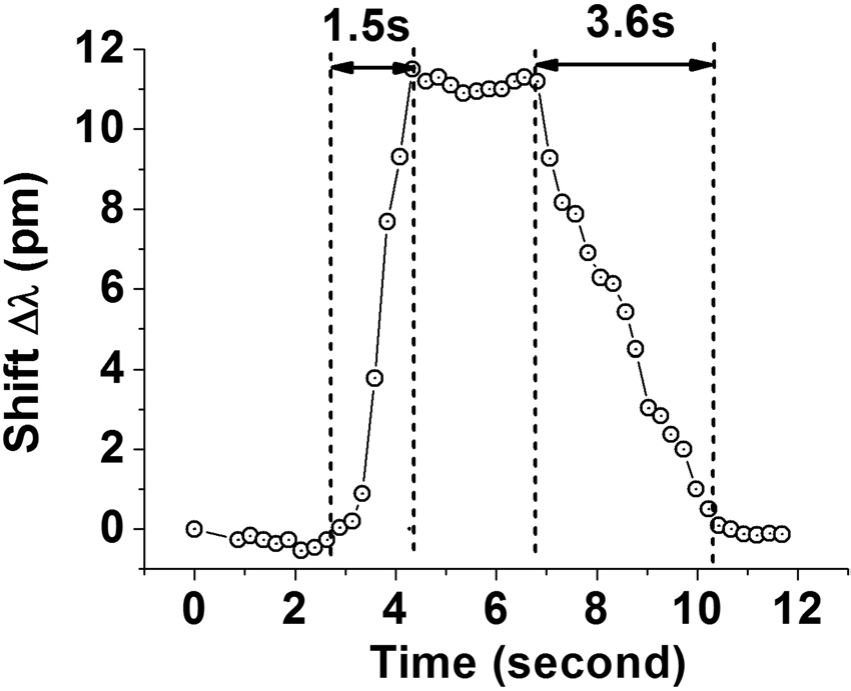
Dynamic response-recovery curve of the sensor to NH_3_ at constant room temperature 23 °C and constant humidity of 50% RH.

The response and recovery times are two important parameters for any sensor. The response time is the time taken by the sensor to reach 90% of the total shift in the resonance wavelength after its exposure to ammonia vapor inside the chamber, while the recovery time is the time taken to reach 90% of the change in wavelength after withdrawing the vapor from the chamber. For the response and recovery time estimations it is not possible to use the optical spectrum analyzer due to its slow wavelength sweep cycles. To capture and process the changes in the WGM transmission spectrum with higher speed we used a tunable laser, photo-detector and the high-speed digital oscilloscope. A customized Labview programme was developed to capture the output screen image of the oscilloscope with a speed of 5 frames per second with each frame containing 1000 data points. The response and recovery times of the sensor were measured in response to NH_3_ concentration of 12 ppb at constant temperature of 23 °C and humidity of 50% RH inside the test chamber. The response time is calculated to be approximately 1.5 seconds, whereas the recovery time was measured to be 3.6 seconds as can be seen from Fig. 11, demonstrating the dynamic response of the sensor based on the oscilloscope image analysis. It should be noted that the response and recovery times include those of the chamber itself and given the volume of the chamber it is very likely that the actual the response times of the sensor are considerably better.

Table 2 below summarizes the performance of several fiber optic ammonia sensors reported in the literature utilizing different physical principles in comparison with our proposed sensor. As can be seen from the table, our proposed sensor offers the detection limit of two orders of magnitude better than any of the optical fiber sensors reported to date.

**Table 2:** Comparative study of performance of various optical ammonia sensors.

Optical technique	Range (ppm)	Response/recovery times (sec)	Detection Limit (ppb)	Ref.
Bromocresol purple coated U-shaped plastic fiber	145–3518	10	10,000	^7^
Silica gel coated micro fiber coupler	0.25–10.4	50/35	5	^23^
Surface plasmon resonance, Ag/Sn *O*_2_	10–100	n/a	154	^24^
Nanostructured dye-doped	12–216	50	5,000	^25^
Mach-Zehnder interferometer using graphene	0–360	0.5	300	^26^
Graphene-coated microfiber Bragg grating	0–100	n/a	200	^27^
**Silica gel coated microsphere (this work)**	**0.0025–0.0123**	**1.5/3.6**	**0.16**	

## Conclusion

A compact optical sensor based on a whispering gallery mode micro resonator for detection of vapor phase ammonia concentration has been proposed and experimentally demonstrated. WGMs are excited in the silica microsphere dip-coated with silica gel and evanescently coupled to an adiabatic fiber tapered. A change in the refractive index of the coating arising due to its exposure to low concentrations of ammonia in air leads to a spectral shift of the WGMs. Two different interrogation methods have been applied to study the sensor’s response to ammonia vapor in two different ranges of concentrations: a wider, low concentrations, range and a narrow range of ultra-low concentrations. The highest sensitivity achieved in our experiments was 34.78 pm/ppm to ammonia concentration using an OSA, and 800 pm/ppm for the ultra-low concentrations of ammonia using a frequency detuning method. Studies of cross sensitivity to humidity have shown a sensitivity 0.3 pm/% RH in the RH range from 30% to 65% at a constant temperature of 23 °C. The proposed sensor offers the advantages of compact size, low cost and high sensitivity. Experimental studies reveal an excellent performance for the proposed sensor in terms of its selectivity to ammonia in the presence of other volatile organic compounds with an ammonia detection limit calculated as 0.16 ppb and a fast response and recovery times in the order of seconds.
